# Meat and Poison

**Published:** 1875-11

**Authors:** 


### MEAT AND POISON.

It is an old saying, and as true as old, that "one man's meat is another man's poison." Not every man can eat and drink with impunity, and digest with facility, whatever is placed before him.

Our ultra radical vegetarian brethren insist that all human beings must eat just such food, prepared in just such a way. The cereals, the fruits, and the vegetables only, are permitted, and all the rest of the catalogue of foods is poison. The bread must be made from coarsely powdered grains or grains not powdered at all. Now we have seen multitudes of people who are made very uncomfortable from eating coarse wheat food. It may be that the stomachs of such persons have been weakened by long use of unnatural foods; but true it is that the articles which prove salutary and wholesome for some, induce very uncomfortable sensations in others. Coarse Graham-bread sours and ferments in the stomach, inducing heartburn and acid conditions, while finer breads are eaten and enjoyed.

The same is true of vegetables. We have known a strong, active man, to whom a fragment of mild, well cooked squash, was like a live coal in the stomach. The same person could eat a whole mince-pie of the richest kind, and feel well after it.

Now what we desire to do, in this matter of food, is merely to explain what is good for the average man. The abnormal man—the man with a depraved or badly tutored appetite—may swallow foods suited to his deranged palate, and feel better than he would if he ate foods known to science as wholesome. There are abnormal natural tastes, too, as well as acquired ones, and these it is folly to war against: they are inherited.

Perhaps most people in eating forget that the object sought is sustenance, support, and not to please the taste, or convey a new and startling sensation to the palate. There was an old gourmand of a king who offered a fortune to any cook who would invent for him a new and appetizing dish. He would eat as long as new dishes were set before him; and so it is no wonder that apoplexy took him off, after one of his debauches.

Nutritious food is always good food, but palatable food is not always good by any means. Some people like pickles, and eat them to excess; but pickles are not good food, because you can not get a pound of flesh out of all the pickles in the world. The same is true of condiments and spices, which have become a necessity to most people. Not that a flavoring of pepper or spice is very harmful, but only that they have no real value considered as supporters of life and repairers of waste.

What we ask sensible people to do is simply this: learn by reading what articles of food are intrinsically valuable; find out by investigation what will best sustain the body in health and strength; and from the list select such as you like best, and get the most comfort and strength from. Then do not have the good foods ruined by bad methods of preparation. Cooks are all-powerful spoilers of food, and can ruin in an hour the food which it has required a hard day's labor to earn.

It would be well if housekeepers would learn precisely WHAT FOOD COSTS. They should understand that in some popular articles consumed as food, there is very little food, and therefore that the money paid for them is chiefly wasted. This fact known, costly articles of small food-value would be used sparingly, and greater attention would be paid to the preparation, in palatable forms, of foods of real value. This course would in time drive such impoverished foods as white flour out of general use; and equally fine but less snowy flour, rich in nutriment, would be substituted for every purpose.

